# Body Position Affects Capillary Blood Flow Regulation Measured with Wearable Blood Flow Sensors

**DOI:** 10.3390/diagnostics11030436

**Published:** 2021-03-04

**Authors:** Andrey A. Fedorovich, Yulia I. Loktionova, Elena V. Zharkikh, Maria A. Mikhailova, Julia A. Popova, Alexander V. Suvorov, Evgeny A. Zherebtsov

**Affiliations:** 1 National Medical Research Center for Therapy and Preventive Medicine of the Ministry of Healthcare of the Russian Federation, Petroverigsky 10, 101990 Moscow, Russia; faa-micro@yandex.ru (A.A.F.); marya.filina-2015@yandex.ru (M.A.M.); 2 Russian Federation State Research Center, Institute of Biomedical Problems of the Russian Academy of Sciences, Khoroshevskoe Highway 76A, 123007 Moscow, Russia; julija.popova@gmail.com (J.A.P.); suvalex@inbox.ru (A.V.S.); 3 Orel State University, Komsomolskaya 95, 302026 Orel, Russia; julya-loktionova@mail.ru (Y.I.L.); ev.zharkikh@gmail.com (E.V.Z.); 4 Optoelectronics and Measurement Techniques, University of Oulu, Erkki Koiso-Kanttilankatu 3, 90014 Oulu, Finland

**Keywords:** wearable blood flow sensors, blood perfusion, laser Doppler flowmetry, ortostatic test, postural changes, body position, blood perfusion in forehead, blood perfusion in wrists, blood perfusion in shins, blood perfusion oscillations, vasomotions

## Abstract

In this study we demonstrate what kind of relative alterations can be expected in average perfusion and blood flow oscillations during postural changes being measured in the skin of limbs and on the brow of the forehead by wearable laser Doppler flowmetry (LDF) sensors. The aims of the study were to evaluate the dynamics of cutaneous blood perfusion and the regulatory mechanisms of blood microcirculation in the areas of interest, and evaluate the possible significance of those effects for the diagnostics based on blood perfusion monitoring. The study involved 10 conditionally healthy volunteers (44 ± 12 years). Wearable laser Doppler flowmetry monitors were fixed at six points on the body: two devices were fixed on the forehead, on the brow; two were on the distal thirds of the right and left forearms; and two were on the distal thirds of the right and left lower legs. The protocol was used to record three body positions on the tilt table for orthostatic test for each volunteer in the following sequence: (a) supine body position; (b) upright body position (+75°); (c) tilted with the feet elevated above the head and the inclination of body axis of 15° (−15°, Trendelenburg position). Skin blood perfusion was recorded for 10 min in each body position, followed by the amplitude–frequency analysis of the registered signals using wavelet decomposition. The measurements were supplemented with the blood pressure and heart rate for every body position analysed. The results identified a statistically significant transformation in microcirculation parameters of the average level of skin blood perfusion and oscillations of amplitudes of neurogenic, myogenic and cardiac sensors caused by the postural changes. In paper, we present the analysis of microcirculation in the skin of the forehead, which for the first time was carried out in various positions of the body. The area is supplied by the internal carotid artery system and can be of particular interest for evaluation of the sufficiency of blood supply for the brain.

## 1. Introduction

The functional state and balance in regulation mechanisms of the cardiovascular system are some of the main factors determining the robustness in a living organism. The evolutionary development of vertebrates in a field of gravitational attraction has led to series of adaptations in the blood supply system. Changes in hemodynamic parameters depending on the position of the body significantly assist the homeostasis in the limbs and essentially in the sufficiency of blood supply for the brain [1]. For the last two decades, laser Doppler perfusion monitoring has become an established technique capable of providing useful diagnostic information about parameters of regulation of the skin blood perfusion. The recent emergence of laser Doppler flowmetry (LDF) as a wearable device has allowed for detailed assessments of individual adaptive capabilities of the blood flow circulation system and can be of particular interest for diagnostics and sport medicine. The next decade is likely to witness a considerable rise of novel optical sensor technologies in wearable sensors, not only to be used as fitness trackers, but to provide clinicians with diagnostic information with better sensitivity and specificity.

Earlier studies have shown significant variability of the cardiovascular system’s parameters (in particular, heart rate and blood pressure) associated with posture and body position [2,3,4]. It has also been shown that parameters of gender and age significantly affect the reactivity of blood flow in response to postural change [5]. It is known that different anatomical parts of the human body demonstrate differences in the blood flow regulation mechanisms [6]. In that respect, regional variability of the effects in microcirculation should also be taken into account. Previous studies reported that the low-frequency mechanisms of microcirculation regulation (endothelial, neurogenic and myogenic) measured with the LDF technique differ between the arm and leg regions under thermoneutral conditions [7,8]. It has also been shown that regulation of microcirculation differs in the leg and forearm under local heating [8]. The regions of glabrous and nonglabrous skin are also reported to have different responses of different types in the parameters of blood perfusion under the type of functional loading [9].

In the study of I. Tikhonova et al., it was found that a postural test (change of supine position to sitting) did not influence the forearm skin blood flow oscillations; they noted a remarkable increase in the respiratory flow and a decrease in the cardiac oscillations in the blood microcirculation in the skin of the legs [10]. The work [11] presents the results of a study of the effects of body position on oxygen consumption ($VO_{2}$) and hemodynamics. It was found that the heart rate, the blood pressure and the product of velocity pressure and oxygen consumption were highest in the sitting position compared to the lying position, and lowest in the lying position on the left side. Narayanan et al. [12] published results on a study of changes in the parameters of blood pressure (BP) and the speed of cerebral blood flow (CBFV) when changing the body position from sitting to vertical in young and old people. It is noted that in young people the linear relationship between blood pressure and the blood flow rate of the middle cerebral artery in stationary sitting conditions changes with orthostatic stress in a wide range of physiological frequencies. Nevertheless, the effects in the parameters of skin microcirculation during changes of the posture and body position were not studied comprehensively, so a lack of systematically conducted research can be identified in this area.

Multi-point measurements using recently developed wearable laser Doppler flowmetry devices [13] can be effectively used for simultaneous recordings of blood perfusion signals from arbitrary anatomical skin sites, thereby providing great potential for finding multiple applications in physiological measurements and medial diagnostics in the near future. One of the challenges that can be mitigated by the use of the distributed measuring system is the difficulty of the high spatial heterogeneity of the LDF signal. Recently, the prototypes of the measuring system have been validated by authors of the work demonstrating the effectiveness of using laser wearable Doppler analyzers for measurements of the parameters of skin blood microcirculation. It has been demonstrated that the sensitivity of the wearable 850 nm VCSEL-basedblood perfusion sensors is sufficient to reliably register physiological changes in skin blood perfusion [14,15,16], including high coherence of blood flow oscillation in the contralateral limbs of healthy volunteers in the basal state and during functional tests [17].

The wireless LDF sensors have been tested in the realm of pre-clinical trials in healthy volunteers of different ages and patients with type 2 diabetes [14,16,18,19]. Additionally, the dynamical changes in the blood perfusion evaluated by LDF and laser speckle contrast imaging techniques were compared, demonstrating that both techniques can be used for the recording of the blood perfusion oscillations [20].

Thus, the use of wearable LDF sensors is promising for both health monitoring, and for evaluating the effectiveness of treatment and monitoring its dynamics. While the multi-point recordings of blood perfusion have demonstrated great promise for the diagnostics of vascular complications, there is a significant gap of knowledge on the effects of the body position during measurements, which introduce systematic impact and additional variability to the recorded signals, which requires accurate systematic studies for the main cases such as measurements while standing upright and in supine position.

Thus far, to the best of our knowledge, no one has systematically studied the effects of postural changes on the skin blood flow by use of wearable LDF sensors as a prime measuring technique. A review identified only one study that used a miniaturized LDF device for the measurements of hemodynamic changes in response to changes of body position [21]. The authors reported a decrease in earlobe microcirculation in response to the squat–standing and the footstool standing tests synchronized with the decrease in blood pressure in subjects. Nevertheless, the mentioned research lacks systematic studies of the effects taking place during the transition of body position from lying supine to standing upright. Thus, the overall aim of this work was to study the reaction of the microcirculation system in skin to changes in body position using the newest wireless wearable measuring platform for the multi-point blood perfusion recordings.

## 2. Material and Methods

**Figure 1:**
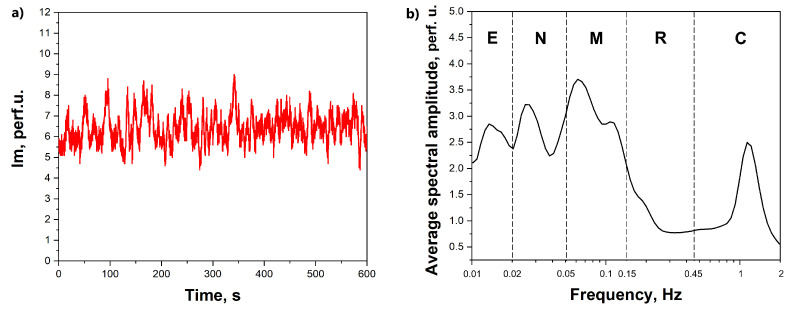
Representative trace of laser Doppler flowmetry (LDF) recordings by the employed measuring system (**a**), the wavelet analysis of the LDF signal with the highlighted frequency ranges for E—endothelial (e, 0.095–0.021 Hz), N—neurogenic (n, 0.021–0.052 Hz), M—myogenic (m, 0.052–0.145 Hz), R—respiratory (r, 0.145–0.6 Hz) and C—cardiac (c, 0.6–2 Hz) regions of blood flow modulation (**b**).

The technique of laser Doppler flowmetry (LDF) measurements with a prototyping system consisting of 6 wireless compact sensors manufactured by SPE “LAZMA” Ltd. (Moscow, Russia) has been applied in this study for the registration of the skin blood perfusion. The LDF method is based on the coherent techniques with the analysis of the laser radiation scattered by moving red blood cells in the living tissue. The output signal of blood perfusion with the LDF method (Figure 1) is a time sequence of an integral parameter that depends on the speed of red blood cells and their concentration in the diagnosed volume.

The distributed measuring system has built-in channels for recording microcirculation blood flow and allows for simultaneous measurements at multiple points of the human body. Every measuring device of the system employs compact VCSELs with an emission wavelength of 850 nm and the power of output of the laser radiation of about 1 mW. Apart from the blood flow measurements, the analyzers were also equipped with a built-in accelerometer to monitor and eliminate possible motion artifacts and a skin temperature sensor.

The object of the study was a cohort of 10 conditionally healthy male volunteers, whose average age was 44 ± 12 years, height 177 ± 6 cm, weight 77 ± 6 kg, BMI 24.5 ± 1.9. All participants were staff testers of the Institute of Biomedical Problems of the Russian Academy of Sciences (IBMP RAS); twice a year they undergo a comprehensive clinical examination for admission to participate in the physiological studies. The main areas of scientific activity of the Institute are research in the fields of space biology, physiology and medicine, which is the reason for the high requirements for the physiological state of the testers. The IBMP RAS Biomedical Ethics Commission has approved the experimental studies, min number 483 dated 3 August 2018, following the rules of the Declaration of Helsinki of 1975, revised in 2013. All volunteers signed informed consent prior to the study. The LDF sensors were located at 6 points on the body: 2 devices were fixed on the forehead above the eyebrows; 2 on the distal third of the outer surface of the forearm (each arm), 2–3 cm proximal to the wrist joint; and 2-in the distal third of the shins along the anterior surface of the tibia, 10 cm proximal to the medial malleolus.

**Figure 2:**
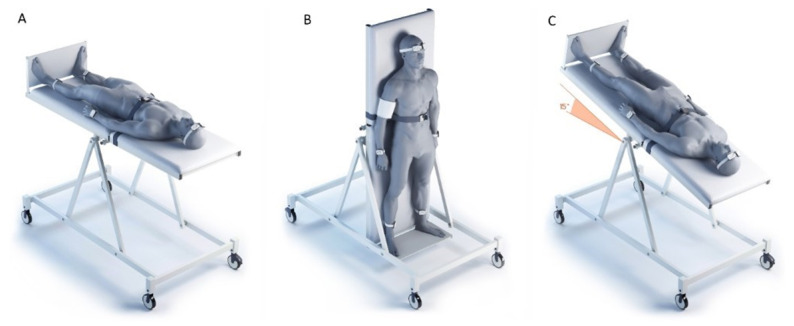
The measurements have been conducted and compared for three distinct body positions on a tilt table: (**A**) supine; (**B**) upright; (**C**) tilted with the feet elevated above the head and an inclination of body axis of 15° (Trendelenburg position).

The studies were carried out in a laboratory with a maintained microclimate (air temperature +23±1 ${}^{{}^{\circ}}$C; humidity 40–60%) in the morning (from 09:00 to 12:00). The studies were carried out in the same order on all subjects (Figure 2)—(1) horizontal position; (2) orthostasis ($+75^{\circ }$); (3) head-down position of the body ($-15^{\circ }$, Trendelenburg position). The study was carried out on a turntable, which was developed and manufactured by the Special Design Bureau of the Institute of Biomedical Problems of the Russian Academy of Sciences. The table has a mechanical drive that allows one to change and fix the angle of inclination of the surface with a step size of $5^{\circ }$ in the range from $-30^{\circ }$ to $+90^{\circ }$ with a maximum speed of position change of up to 20 ${}^{\circ }$/s. The table is equipped with a leg rest, and chest and knee safety belts. Transfer of subjects from horizontal position to orthostasis took 7–10 s, from orthostasis to Trendelenburg position—9–12 s. Cutaneous perfusion was recorded for 10 min at each body position. The adaptation of the subjects to the horizontal position lasted 10–15 min, during which time the sensors were fixed and the research equipment was adjusted. During the transition to orthostasis and the Trendelenburg position, the registration of cutaneous perfusion began after 2 min of adaptation to the new body position. Immediately before the change in body position, hemodynamic parameters were recorded with an automatic tonometer “OMRON M10-IT” (OMRON HEALTHCARE Co, Ltd., Kyoto, Japan) on the right hand, due to the design features of the turntable—a technical “pocket” for placing additional research equipment is located on the right. The temperature of the skin in each area of the study was monitored continuously throughout the entire study by built-in thermal sensors. The protocol was used for recordings in three body positions for each volunteer (Figure 2): (a) horizontal body position; (b) vertical position of the body (head at the top); (c) head tilted down (15° from the horizontal, Trendelenburg position).

The applied combination of the Trendelenburg position and orthostatic probe makes it possible to characterize the functional reserve of the blood circulatory system for the volunteers, and to correlate the adaptations of peripheral hemodynamics to the body position changes. The Trendelenburg position is known to be an effective method to change cerebral perfusion, and to fill and stretch the upper central veins and the external jugular vein [22].

The measuring procedure was composed of several stages. One basal recording of blood perfusion for every body position took 10 min; then the blood pressure was measured. Thus, for each spatial position, three pairs of measurements were recorded at the corresponding symmetrical points on the forehead, wrists and shins.

The amplitude–frequency characteristics of the skin perfusion oscillations were calculated using the mathematical apparatus of the wavelet transform. The wavelet spectrum of the signal was calculated according to the following expression:

(1) $$W\left(s,\tau \right)=\frac{1}{\sqrt{s}}\int_{-\infty }^{\infty }x\left(t\right)\psi^{*}\left(\frac{t-\tau }{s}\right)$$

where *x(t)* is a sample of the signal, τ is time index, *s* is scaling factor, * means complex conjugation. As a core wavelet, Morlet wavelet function $\psi \left(t\right)=e^{2\pi it}\cdot e^{-t^{2}/\sigma }$ was choosen with decay parameter σ=1.

The time-averaged amplitude of vasomotions was assessed by the maximum values (Ai) in the corresponding frequency ranges for endothelial (e, 0.095–0.021 Hz), neurogenic (n, 0.021–0.052 Hz), myogenic (m, 0.052–0.145 Hz), respiratory (r, 0.145–0.6 Hz) and cardiac (c, 0.6–2 Hz) regions of blood flow modulation [23] (Figure 1b). The level of cutaneous perfusion (Im) and the amplitude of the units of modulation of microcirculation (Ai) were assessed as quantitative parameters measured in arbitrary (perfusion) units (p.u.). The wavelet analysis has been implemented in the MATLAB software environment. The LDF signals in this particular study were not a subject of pre-processing or filtering before the analysis. The statistical analysis was performed in Origin Pro 2019b (vers. 9.65) software. Due to the limited size of the sample, a non-parametric Mann–Whitney U test was used for the check of statistical significance of differences.

## 3. Results

### 3.1. Measurements Conducted on Wrists

**Figure 3:**
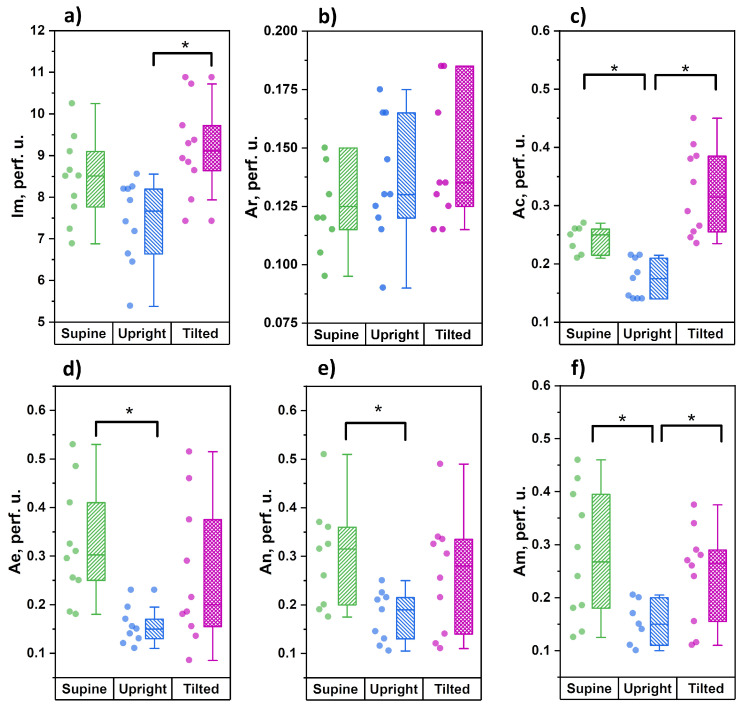
Analysis of average blood perfusion parameters on the wrists for three tested body positions: supine, upright and tilted (Trendelenburg position): (**a**) average blood perfusion; (**b**) cardiac oscillations; (**c**) respiratory oscillations; (**d**) endotelial oscillations; (**e**) neurogenic oscillations; (**f**) myogenic oscillations (* the significance of a difference between values was confirmed with *p* < 0.05 using the the Mann–Whitney test).

The results of the amplitude analysis of cutaneous perfusion in the skin of the wrists are shown in Figure 3.

From the data obtained, it can be seen that during the transition from the horizontal position to orthostasis, the level of cutaneous perfusion has an insignificant tendency to decrease, which is accompanied by significant decreases in the amplitude of cardiac oscillations in blood flow and the amplitude of vasomotions of all tone-forming mechanisms of microcirculation—endothelial, neurogenic and myogenic. During the transition from orthostasis to the Trendelenburg position, the level of skin perfusion significantly increased, which was accompanied by significant increases in the amplitudes of cardiac and myogenic oscillations. There were no significant differences between the Trendelenburg position and the horizontal position for any of the analyzed parameters. The level of perfusion and the amplitude of the cardiac fluctuations both have a clear tendency to increase, but we did not find significant differences.

### 3.2. Measurements on Lower Legs

**Figure 4:**
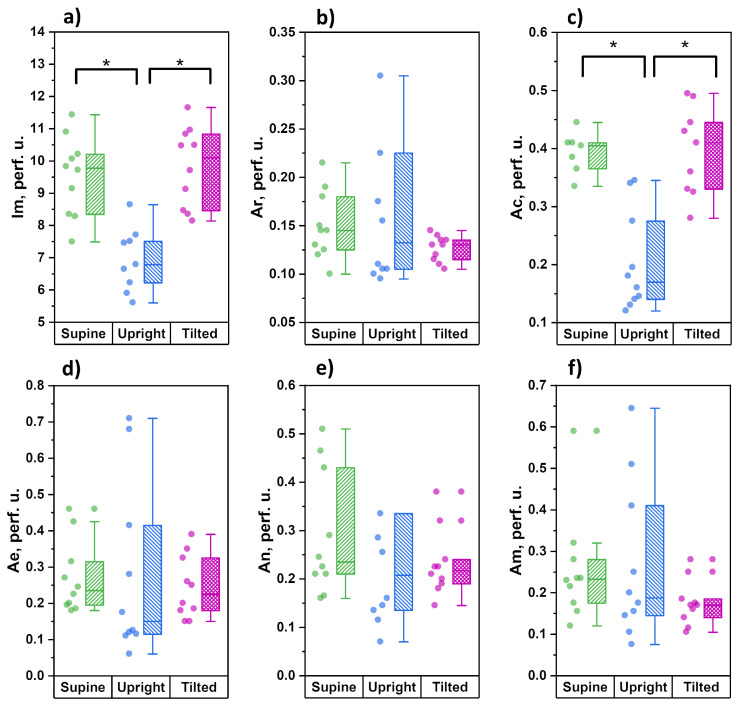
Analysed blood perfusion parameters measured on the shins for three body positions: supine, upright and tilted (Trendelenburg position): (**a**) average blood perfusion; (**b**) cardiac oscillations; (**c**) respiratory oscillations; (**d**) endotelial oscillations; (**e**) neurogenic oscillations; (**f**) miogenic oscillations (* the significance of a difference between values was confirmed with *p* < 0.05 using the Mann–Whitney test).

Figure 4 demonstrates the distribution of the studied parameters during measurements in the lower third of shin.

The parameters of microcirculatory blood flow demonstrated a significant decrease in the average level of tissue perfusion and the amplitude of cardiac oscillations during the transition to orthostasis. During the change from orthostasis to the Trendelenburg position, these parameters significantly increased and were comparable with those measured in the horizontal position. In contrast to the measurements conducted in forearms, the functional state of the tone-forming mechanisms of microcirculation modulation in shins (parameters Ae, An and Am) did not demonstrate any significant changes during all three stages of the study.

### 3.3. Measurements on the Forehead

**Figure 5:**
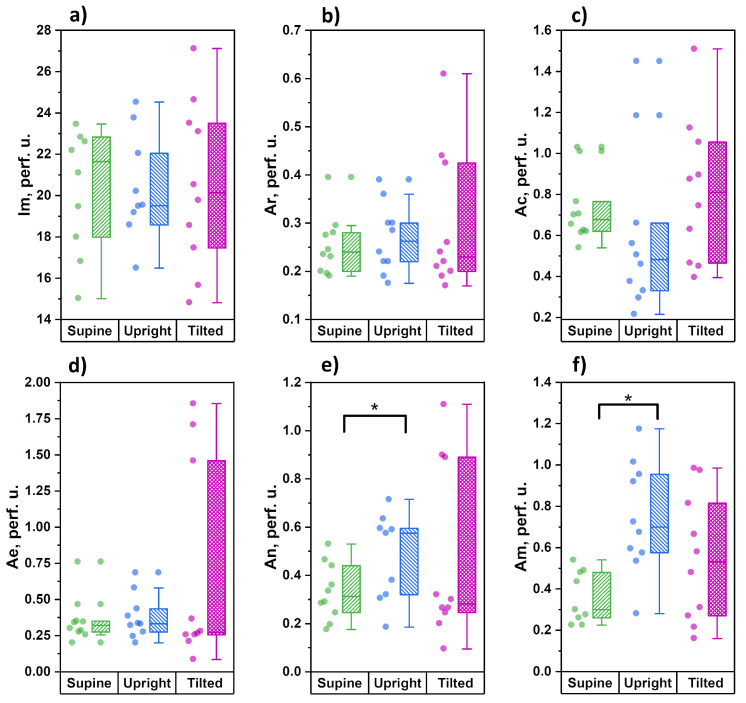
Analysis of blood perfusion parameters in the skin of the brow of the forehead for three tested body positions: supine, upright and tilted (Trendelenburg position): (**a**) average blood perfusion; (**b**) cardiac oscillations; (**c**) respiratory oscillations; (**d**) endotelial oscillations; (**e**) neurogenic oscillations; (**f**) miogenic oscillations (* the significance of a difference between values was confirmed with *p* < 0.05 using the Mann–Whitney test).

Figure 5 shows the results of the measurements of the cutaneous blood perfusion dynamics on the forehead.

During measurements on the forehead, we did not register any significant changes in the index of microcirculation caused by postural changes. With unchanged tissue perfusion, however, significant increases in the amplitudes of neurogenic and myogenic oscillations were recorded when changing from a supine to an upright position, which is the opposite of the results obtained in the shins. Despite the presence of a tendency towards moderate increases in the amplitudes of endothelial and neurogenic oscillations in the Trendelenburg position, these changes did not reach statistically significant levels.

### 3.4. Blood Pressure and Heart Rate

**Figure 6:**
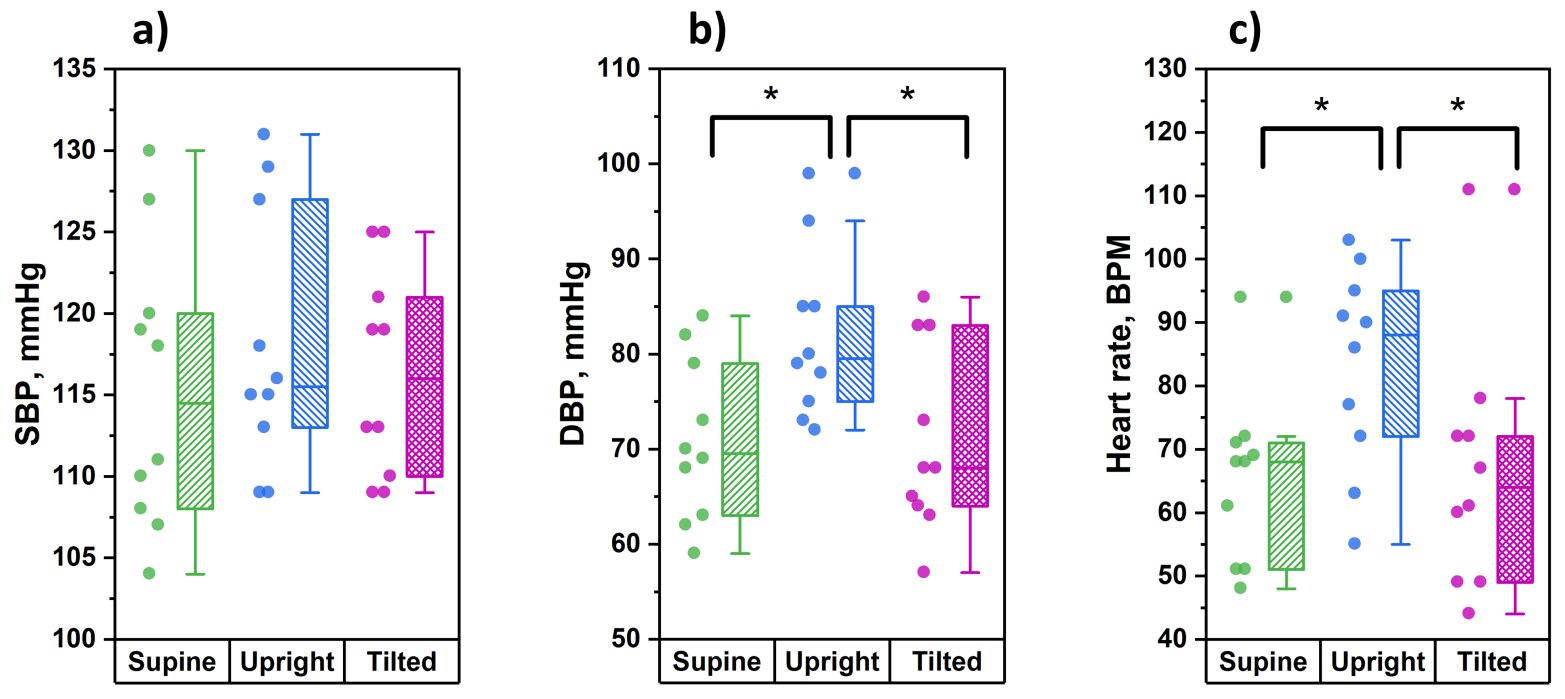
Analysis of parameters of blood pressure and hear rate measured in the tested body positions: supine, upright and tilted (Trendelenburg position): (**a**) systolic blood pressure; (**b**) diastolic blood pressure; (**c**) heart rate (* the significance of a difference between the values was confirmed with *p* < 0.05 using the Mann–Whitney test).

Before each stage of the study, for every tested subject the parameters of blood pressure and heart rate were recorded via the right arm. The results of these measurements are shown in Figure 6.

From the data obtained, it can be seen that in the position of orthostasis, significantly higher values of diastolic pressure and heart rate were observed compared to the supine and the Trendelenburg positions. At the same time, the values of systolic blood pressure did not undergo significant changes during postural changes.

## 4. Discussion

The value of the skin microvascular bed as an object of research for identifying the patterns of the cardiovascular system functioning is under discussion nowadays. In a review work, based on the results of LDF amplitude–frequency wavelet analysis, Martini R. and Bagno A. showed that changes in the parameters of the skin microcirculation are detected in the widest range of diseases [24]. This includes studies of diabetes complications [25], peripheral arterial disease [26] and arterial hypertension [27] among other conditions.

It is known that the microvascular bed of the skin is not subject to baroreflex regulation [28,29], and the results we obtained on the forearm are very interesting. During the transition to orthostasis, when the measurement area was below the height of the heart, we noted decreases in the amplitudes of endothelial, neurogenic and myogenic vasomotions. A decrease in the vasomotions’ amplitude indicates a decrease in the lumen size of resistive precapillary arterioles, which can be regarded as an increase in the vascular tone. It can be assumed that a decrease in the lumen of resistive precapillary arterioles leads to a decrease in the amplitude of cardiac oscillations in microvessels, which, in turn, indicates a decrease in arterial blood flow to the capillaries. A decrease in the amplitude of cardiac oscillations at the level of precapillary arterioles can be caused by several mechanisms: (1) An increase in hydrostatic pressure in the venular link of the vascular bed amidst the difficulty in the blood outflow from the capillaries leads to an increase in the capacitive vessels tone, which, through the mechanisms of venulo-arteriolar communication, can lead to an increase in the tone of the bringing arterioles [30]. (2) Activation of the sympathoadrenal system due to weakening of the depressor effects on it from the baroreceptors of the carotid sinus. Amidst that, the subjects showed a significant increase in diastolic blood pressure and heart rate. We did not find a significant correlation between these parameters, which may have been due to a small sample size, but it can be assumed that there is a relationship between the amplitude of vasomotions of the tone-forming mechanisms of skin microvessels and the level of blood pressure. If this hypothesis is correct, then the LDF technique can be a useful additional tool for interpreting the results of 24-h blood pressure monitoring when patients are in an upright position for most of the day, and for monitoring the functional state of resistive skin microvessels when prescribing antihypertensive therapy.

When the head is lowered 15° below the horizontal line, the measurement point on the forearms is slightly above the heart level, which does not affect the functional state of the tone-forming regulatory mechanisms of skin microcirculation in the upper extremities. Amidst this, there is a significant increase in the amplitude of the cardiac oscillations, which can be explained by the opposite mechanisms observed in orthostasis: (1) the precapillary arterioles’ tone is restored through the mechanisms of venulo-arteriolar communication against the background of a decrease in pressure in the venous vessels; (2) a decrease in the activity of the sympathoadrenal system amidst the restoration of the depressor activity of the carotid sinuses. An increase in the arterial blood inflow into the microvasculature is accompanied by a significant increase in the level of tissue perfusion (Figure 3a). For the lower extremities, a change in body position leads to a decrease in the amplitude of cardiac oscillations (a decrease in inflow), and a corresponding decrease in the level of tissue perfusion without changing the activity of tone-forming mechanisms at the level of resistive precapillary arterioles. This is most likely due to regional features of the tissue perfusion regulation of the skin in the legs and is of a compensatory nature aimed at maintaining nutritive blood flow in conditions of decreased tissue perfusion. In a non-physiological position for the legs, when they are above the heart, the outflow of venous blood is significantly facilitated, but the perfusion pressure decreases. Amidst this, we see an insignificant tendency towards a decrease in the amplitude of neurogenic and myogenic vasomotions (increased tone), which can also be regarded as a compensatory response aimed at maintaining perfusion pressure in the skin capillaries.

The results of the study of skin perfusion in the forehead can be of particular interest in connection with the brain’s blood supply. It is known that the scalp receives nutrition from the external carotid artery system, and only the skin of the forehead is supplied with blood from the a.supratrochlearis and a.supraorbitalis, which are the final branches of the supraorbital arteries that are part of the internal carotid artery system [31]. The researchers’ interest in the basin of the a.supraorbitalis is due to the fact that disorders of microcirculatory blood flow in the eye area (fundus and bulbar conjunctiva) are associated with various variants of cerebral circulatory disorders [32,33,34]. As was shown in the pilot study [35], the nature of skin microcirculation in the forehead significantly differs in the level of skin perfusion and in the activity of regulatory mechanisms, depending on the side and volume of ischemic brain damage, and during thrombolytic therapy, these parameters showed significant changes.

The higher level of average blood perfusion in skin of the forehead, relative to the skin of the upper and lower extremities, and the stability of cutaneous blood perfusion in any position of the body (Figure 5a), draw attention. This may indicate a high potential of the mechanisms of autoregulation of cerebral blood flow. Significant changes in the regulatory mechanisms at the level of precapillary arterioles are observed only in orthostasis and are expressed in an increase of amplitude of neurogenic and myogenic vasomotations. When the head is higher than the heart, the neurogenic and myogenic mechanisms of microvascular tone-forming are reduced.

In the Trendelenburg position, when not only the outflow of venous blood from the head is hindered, but also the pressure in the arterial bed increases, the functional state of the tone-forming mechanisms changes in a very wide range. We assume that this is due to the high potential of the mechanisms of regulation of cerebral hemodynamics. For the tone-forming mechanisms of microcirculation regulation (Ae, An and Am), changing their functional activity according to the principle of positive and negative responses, modulates the volume and speed of arterial blood flowing to the capillaries (Ac) to the optimal values for transcapillary exchange in the vascular volume at the time, and in our study—depending on the position of the body in space.

The facial skin, as an object of research, is also interesting due to its features of innervation. The system of innervation of skin microvessels is mainly represented by somatic sensitive (afferent) and vegetative sympathetic (efferent) systems of regulation. The direct involvement of the parasympathetic nervous system in the regulation of cutaneous microvessels is considered proven only for the skin of the face [32,36]. Thus, the study of microcirculatory blood flow in the area of the facial skin opens up opportunities for studying almost all mechanisms of neurogenic control of the vasomotor activity of resistive microvessels at the opposite end to the heart pole of the great circle of blood circulation [37].

## 5. Conclusions

The novel wearable sensors implementing the measuring principles of laser Doppler and dynamic light scattering techniques for blood perfusion monitoring significantly extend the capabilities of researchers and practical clinicians in terms of monitoring parameters of skin blood perfusion. The analysis of signals recorded by the wearable LDF devices can be effectively realized using machine learning algorithms. Nevertheless, practical applications of the sensors for medical diagnostics require taking into account a considerable number of details. Some of the factors greatly influencing the patterns of the blood perfusion behavior are the posture and body position. In this study, we demonstrate what kind of relative changes can be expected in average perfusion and blood flow oscillations during postural changes being measured on skin of the limbs and on the brow of forehead. We show the importance of taking into account the position of the body in space during the monitoring of physiological parameters interrelated with blood perfusion. Presented findings of the amplitude–frequency analysis of LDF signals measured in different body positions confirm the early promise of the measurement technique for the orthostatic test and diagnostic procedures based on it, and more specifically, for the use of this particular type of wearable device together with the physiological tests. The results obtained can be of particular interest for the development of new protocols for the study of microcirculation, including those related to daily monitoring. The vast majority of modern fitness trackers use the method of photoplethysmography to record physiological parameters. In this and previous works, we have shown that the LDF method can also be of significant interest for wearable applications, opening up new opportunities for the diagnostics of the microcirculation and cardiovascular systems.

## 6. Patents

1. E.A. Zherebtsov, I.O. Kozlov, A.I. Zherebtsova, E.V. Zharkikh, Y.I. Loktionova and A.V. Dunaev, “Software for data analysis of multi-channel wearable device for recording the level of capillary blood flow.” Software patent RU 2019665950 (2019).

2. I.O. Kozlov and E.A. Zherebtsov, “Software for recording the distribution of blood perfusion by Doppler shift frequencies.” Software patent RU 2019616389 (2019).

## Data Availability

The data presented in this study are available on request from the corresponding author.
